# ‘An ideal service for glaucoma would be…’

**Published:** 2014

**Authors:** Fatima Kyari

**Affiliations:** Ophthalmologist/Senior Lecturer: College of Health Sciences, University of Abuja, Nigeria.

**Figure:**
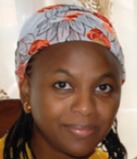
Fatima Kyari

This article proposes a ‘top-down’ approach to developing glaucoma services. To do this, good evidence, gathered through research, is needed about the following:

- The prevalence of different types of glaucoma in the population (as open-angle and angle-closure glaucoma are managed differently).
- The age and socio-economic status of the local population, as well as any biomedical/metabolic or genetic factors that might predispose them to glaucoma. This makes it possible to identify the high-risk groups.
- The local community's knowledge, beliefs and health-seeking behaviour with regards to eye disease.
- The expectations and perceptions of patients and family members.
- The best treatment options (based on randomised controlled clinical trials and outcomes studies), which take into account the local realities and patients' preferences.

All of the above can be used to develop efficient, streamlined services that will encourage patients to come back for long-term care and follow-up, which is essential.

## Suggested steps

The initial focus should be on developing good quality sub-speciality services at the tertiary level, followed by strengthening of secondary eye care (at district level) and then implementation of strategies for the early detection of glaucoma. There should be clear guidelines for referral (in both directions) between tertiary and secondary levels, and from the community to the secondary level once early detection strategies are implemented.

If this approach is ignored, early detection and diagnosis will create false expectations – and eventually disappointment – when patients are told nothing can be done about their diagnosis due to inadequate services at secondary or tertiary level. This will lead to a loss of faith in the eye care service.

The fives steps in this approach are described below.

### Step 1. Strengthen tertiary eye care units to provide a good standard of glaucoma services

**Figure:**
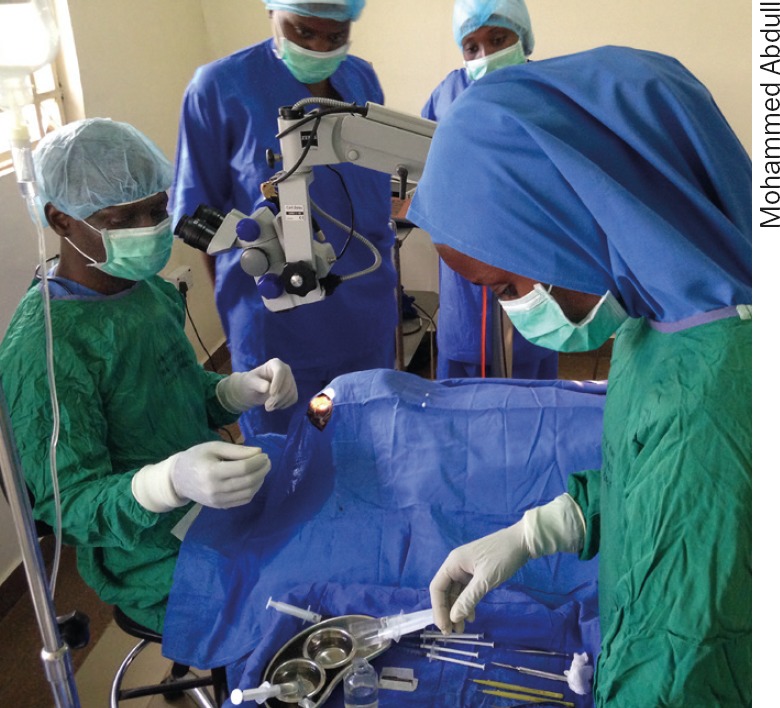
Glaucoma care requires skilled personnel who can work in teams

It must be possible for patients to undergo all the necessary investigations during a single visit. This will reduce costs to patients and encourage them to come back for follow-up visits. The following are also needed at tertiary level:

- **Equipment.** The hospital should be equipped with the appropriate diagnostic and therapeutic equipment.
- **Skilled personnel.** The glaucoma team should consist of glaucoma sub-specialists, general ophthalmologists, optometrists, ophthalmic nurses, counsellors, equipment technicians and other allied eye care providers. The team should be trained to provide accurate diagnosis and prompt, appropriate management with a choice of medical, laser or surgical treatment. Personnel should be able to monitor disease progression and institute treatment using clear clinical guidelines. Task sharing may be required, such as training nurses or technicians to assess visual fields, to take optic disc images or to counsel patients so that clinicians have time to focus on management decisions.
- **Information management.** There should be robust health management information systems and reliable management of medical records to ensure follow-up and monitoring of disease progression.

### Step 2. Strengthen secondary centres (at district level) to manage less complex cases

- There should be a robust referral and feedback system between the tertiary centre and the secondary centres.
- Protocols for ocular examination and glaucoma diagnosis and management should be in place.
- Non-complex glaucoma cases should be managed at the secondary centres. Additionally, patients that had surgery or laser treatment at the tertiary centre can be referred back to the secondary centres for long-term care and follow-up.

### Step 3. Develop glaucoma case-detection strategies at the secondary and primary levels

For example, everyone aged 30 (or 40) years and above who seeks eye care (e.g. with presbyopia or refractive errors) and for driving tests, could be offered a comprehensive eye examination, including optic disc assessment and intraocular pressure measurement, with confirmation of glaucoma diagnosis by visual field testing.

### Step 4. Provide low vision and rehabilitation services

Glaucoma is the commonest cause of functional low vision in Nigeria.^1^ Providing low vision services for glaucoma patients could therefore enhance their functional vision and quality of life.

If a high proportion of the glaucoma patients who come forward are already blind, community-based rehabilitation (CBR) should be an integral part of the glaucoma service provided.

### Step 5. Increase awareness of glaucoma among policy makers and the public

Develop good working relationships with people responsible for health policy, whether at a local or national level. Emphasise that glaucoma is a major cause of irreversible blindness that could potentially be avoided. Encourage policy makers to create supportive policies: for example, to enhance the availability of affordable glaucoma medication and laser treatment at an affordable cost.

A public health awareness campaign for glaucoma should only be instituted when a good glaucoma service is in place. The campaign should be based on local beliefs, attitudes and behaviour, and should make use of suitable communication channels. For example:

- placing posters in public areas
- giving talks and handing out leaflets in hospital waiting rooms
- working with local organisations
- using the media (e.g. radio or television programmes and newspaper articles).
